# Glucose-lowering effects of orally administered superoxide dismutase in type 2 diabetic model rats

**DOI:** 10.1038/s41538-022-00151-5

**Published:** 2022-08-20

**Authors:** Jingke Guo, Hangqi Liu, Dan Zhao, Chaoyi Pan, Xuepu Jin, Yujia Hu, Xiaolu Gao, Pingfan Rao, Shutao Liu

**Affiliations:** 1 Institute of Biotechnology, Fuzhou University, Fuzhou, 350108 China; 2 Zhicheng College, Fuzhou University, Fuzhou, 350002 China; 3 SIBS-Zhejiang Gongshang University Joint Centre for Food and Nutrition Sciences, Zhejiang Gongshang University, Hangzhou, 310012 China

**Keywords:** Gastroenterology, Physiology

## Abstract

Superoxide dismutase (SOD) is an enzyme found in most food sources, might be a candidate to reduce oxidative damage to intestinal barrier, thereby ameliorating the vicious circle between hyperglycemia and the oxidative damage. Here we report the oral administration of SOD, liposome-embedded SOD (L-SOD), and SOD hydrolysate to type 2 diabetic model rats to confirm this hypothesis. Oxidative damage severity in model rat intestine was indicated by malondialdehyde level, GSSG/GSH ratio, and antioxidant enzyme activity. The damage was significantly repaired by L-SOD. Furthermore, blood glucose and related indexes correlated well not only with oxidative damage results but also with indexes indicating physical intestinal damage such as colon density, H&E staining, immunohistochemical analysis of the tight junction proteins occludin and ZO-1 in the colon, as well as lipopolysaccharide and related inflammatory cytokine levels. The order of the magnitude of the effects of these SOD preparations was L-SOD > SOD > SOD hydrolysate. These data indicate that orally administered SOD can exhibit glucose-lowering effect via targeting the intestine of diabetic rats and systemic lipopolysaccharide influx.

## Introduction

The prevalence of diabetes is increasing sharply with the improvement of living standards and global changes in lifestyle. The complications of diabetes caused by hyperglycemia, the main syndrome of diabetes, continue to be a large and increasing global health burden^1,2^. Furthermore, there are problems with existing drugs, such as treatment failure, gastrointestinal side effects, weight gain, and/or hypoglycemia^3^. Therefore, it is urgent to develop alternative glucose-lowering approaches for diabetes.

Recently, dysfunction^4^ of the intestinal barrier^5^ has been suggested to play an important role^6^ in the pathogenesis of diabetes^7^. Thaiss et al. pointed out that hyperglycemia, but not the microbiome, drove intestinal barrier dysfunction and dissemination of bacterial products to the systemic circulation in diabetes^4^. It was also previously reported that the systemic influx of lipopolysaccharide (LPS), a potential pro-inflammatory product from Gram-negative bacteria in the intestine, was increased in the case of intestinal barrier dysfunction^8^. LPS, also known as endotoxin, not only induces systemic inflammation^9^ but is also associated with insulin resistance (IR) and the progression of diabetic complications^10^. These publications indicate that the hyperglycemia of diabetes forms a positive feedback cycle via the systemic influx of LPS from a damaged intestinal barrier. Therefore, dietary bioactive agents targeting the intestinal barrier might be an alternative glucose-lowering approach in diabetes^6,11^.

Regarding the exact reason that hyperglycemia drives the dysfunction of the intestinal barrier, overproduction of superoxide may be the primary issue. It is reported that hyperglycemia in diabetes induces overproduction of superoxide by the mitochondrial electron transport chain^12^. Recently, Guo et al. revealed that mitochondria-producing superoxide could transmit to the extracellular matrix^13^. This overproduced superoxide is hypothesized to cause oxidative stress and damage in intestinal tissue^14^. Based on this hypothesis, the specific catalytic scavenger of superoxide^15^, superoxide dismutase (SOD), which is an enzyme found in most food sources, especially in marine phytoplankton, melons, cruciferous vegetables, is an ideal candidate to alleviate the syndromes of diabetes by reducing the oxidative damage and destruction of the intestinal barrier, thereby ameliorating the vicious circle of hyperglycemia. This would depend on targeting the SOD to the intestine.

Cu/Zn SOD is an unglycosylated homodimer (32 kDa)^16^. SOD was found to be stable between 20–40 °C temperature and pH ranging from 6.0 to 9.0^17^. Orally administered SOD may be a source of intestine-targeted SOD^18^. However, the effect of orally administered SOD on diabetes has not been generally accepted so far, because SOD taken orally degrades in the digestive tract and is not bioavailable for the intestine^19^. However, Regnault et al. suggested that a small amount of SOD remained in the digestive tract and was subsequently bioavailable in the bloodstream after oral administration^20^. Our group preliminarily found that orally administered SOD exhibited a substantial glucose-lowering effect in alloxan-induced diabetic rats^21^.

Because liposomes can increase the tolerance of proteins to digestive enzymes^22^, liposome-embedded SOD (L-SOD) might exhibit higher intestinal bioavailability than natural SOD. In fact, L-SOD was found in our lab to retain 85.84% of enzymatic activity even after being treated in simulated gastric fluid for 4 h, indicating that L-SOD was resistant to potential degradation by gastric fluid. Therefore, L-SOD was used in this work to investigate the possibility of the above-mentioned novel glucose-lowering effect of orally administered SOD, by comparison with natural SOD and SOD hydrolysate. SOD hydrolysate in this work is the product of SOD treated with pepsin for 4 h, with no remaining intact SOD molecule and no detectable enzymatic activity of SOD. These three preparations of SOD represent samples with different SOD enzymatic activities targeting the intestine. We hypothesized the orally administered SOD could exhibit a glucose-lowering effect in diabetes via a direct decrease of oxidative damage in the intestine. If this hypothesis is correct, this work could help to elucidate the function of the intestinal barrier as the target of orally administered SOD in diabetes, thus laying a foundation for the application of orally administered SOD as a novel functional food approach in the control of hyperglycemia in diabetics.

## Results

### Confirmation of oxidative stress in the intestine of T2D model rats and the antioxidant effect of SOD samples

**Fig. 1:**
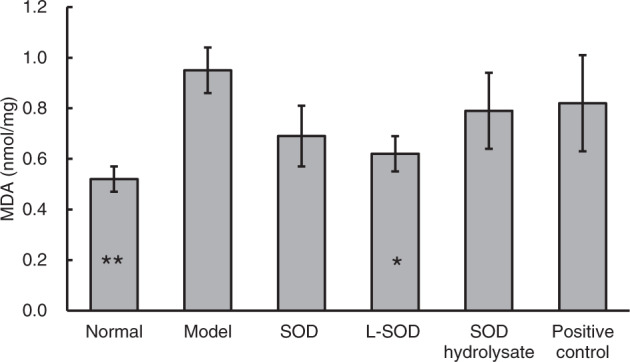
MDA content in the colons of T2D model rats after 4-week intervention. The data are presented as mean ± SD, *n* = 6 each group. *P < 0.05, **P < 0.01 vs. model group.

**Fig. 2:**
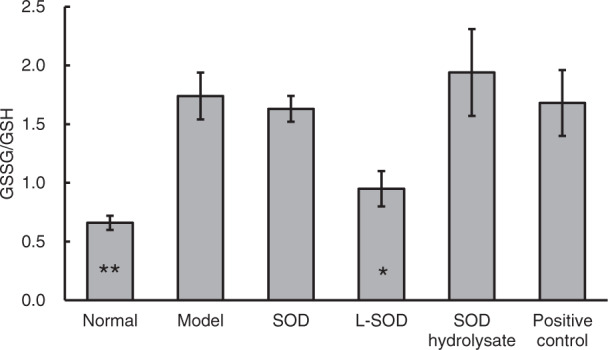
GSSG/GSH in the colons of T2D model rats after 4-week intervention. The data are presented as mean ± SD, *n* = 6 each group. *P < 0.05, **P < 0.01 vs. model group.

To verify the presence of oxidative stress in the intestine of type 2 diabetic (T2D) model rats and to determine if orally administered SOD reduces this oxidative stress, we measured malondialdehyde (MDA), the ratio of oxidized glutathione to reduced glutathione (GSSG/GSH), and antioxidant enzymatic activity of SOD, catalase (CAT), and glutathione peroxidase (GPx) in the colons of normal, model, and intervention (SOD, L-SOD, SOD hydrolysate, or metformin) groups of T2D model rats. MDA is the product of lipid peroxidation, and GSSG/GSH is the ratio of GSSG to GSH. Both indexes reflect the state of oxidative stress. As shown in Fig. 1, the MDA content in colons of the model group of T2D rats was significantly higher than in the normal group, by 82.69% (*P* < 0.01), while the MDA in the L-SOD group was significantly lower than in the model group, by 34.74% (*P* < 0.05). The GSSG/GSH ratio (Fig. 2) of the model group (1.74) was significantly higher than that of the normal group (0.66) (*P* < 0.01), but the value in the L-SOD group (0.95) was significantly lower than in the model group (*P* < 0.05). There was no significant difference between the MDA or GSSG/GSH values of the SOD hydrolysate group, positive control group, and model group (*P* > 0.05). Compared with the model group, the values of MDA and GSSG/GSH in the SOD group exhibited decreasing trends, although this was not significantly different (*P* > 0.05).

Meanwhile, the antioxidant enzymatic activities of SOD, CAT, and GPx were measured in colonic tissues to evaluate further the status of oxidative stress in the intestine (Table 1). SOD, CAT, and GPx in the colons of the L-SOD group were significantly higher than in the model group, by 8.92% (*P* < 0.05), 67.13% (*P* < 0.05), and 46.10% (*P* < 0.05), respectively; and those values in the SOD group were higher than in the model group, by 1.57% (*P* > 0.05), 53.15% (*P* < 0.05), and 45.50% (*P* < 0.05), respectively. We observed that those values in the model T2D group of rats were significantly lower than those in the normal group, by 15.65% (*P* < 0.01), 43.25% (*P* < 0.01), and 46.99% (*P* < 0.01), respectively. These results indicated that there was substantial oxidative stress in the colons of T2D model rats, whereas SOD and L-SOD significantly reduced this oxidative stress, and the antioxidant effect of L-SOD was greater than that of SOD. SOD hydrolysate did not play a significant antioxidant role in the colonic tissue.

**Table 1:** Antioxidant enzymatic activities of SOD, CAT, and GPx in colon tissues.

Group	SOD (U/mg)	CAT (U/mg)	GPx (U/ml)
Normal	781.57 ± 28.05**	2.52 ± 0.18**	578.51 ± 47.02**
Model	659.34 ± 14.21	1.43 ± 0.19	306.67 ± 26.75
SOD	669.66 ± 34.66	2.19 ± 0.25*	446.21 ± 36.31*
L-SOD	718.10 ± 24.77*	2.39 ± 0.29*	448.05 ± 27.49*
SOD hydrolysate	643.36 ± 46.07	1.26 ± 0.39	355.48 ± 30.81
Positive control	681.84 ± 44.69	1.72 ± 0.18	460.46 ± 55.29*

The data are presented as mean ± SD, *n* = 6 each group. *P < 0.05, **P < 0.01 vs. model group.

### Effect of orally administered SOD samples on the blood glucose and related indexes of T2D model rats

**Fig. 3:**
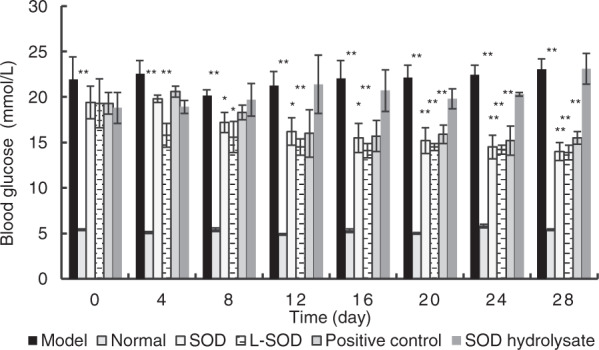
Changes in blood glucose in T2D model rats during 4-week intervention. The data are presented as mean ± SD, *n* = 6 each group. *P < 0.05, **P < 0.01 vs. model group.

As shown in Fig. 3, at the end of the 4-week (28-day) intervention, the blood glucose levels of the SOD group, L-SOD group, and positive control group were significantly lower than that of the model group (*P* < 0.01). Moreover, the blood glucose level of the L-SOD group decreased steadily with a higher rate than those of the SOD group and positive control group during the 4-week intervention, although it reached a plateau after 16 days. However, there was no statistical difference between the SOD hydrolysate group and the model group (*P* > 0.05).

T2D is associated with dyslipidemia and increased lipid peroxidation^23^. Therefore, a decrease of total cholesterol (TC), triglycerides (TG), and low-density lipoprotein cholesterol (LDL-C), and an increase of high-density lipoprotein cholesterol (HDL-C) are important measures in the treatment of diabetes. As shown in Table 2, after 4 weeks of intervention, the levels of TG and LDL-C in the L-SOD group were significantly lower than in the model group, by 34.76% and 33.92%, respectively (*P* < 0.01); the TC level decreased significantly, by 39.39% (*P* < 0.05), compared with the model group; conversely, HDL-C very significantly increased, by 72.42% (*P* < 0.01). The SOD group also exhibited significant improvement, but none of these measures of blood lipids in the SOD hydrolysate group showed any significant difference compared to the model group (*P* > 0.05).

**Table 2:** Fasting plasma lipid levels after 4-week intervention.

Group	TG (mmol/L)	TC (mmol/L)	HDL-C (mmol/L)	LDL-C (mmol/L)
Normal	1.21 ± 0.12**	2.18 ± 0.20*	4.62 ± 0.13**	1.21 ± 0.11**
Model	2.33 ± 0.13	3.63 ± 0.47	2.62 ± 0.40	2.83 ± 0.18
SOD	1.74 ± 0.07**	2.15 ± 0.18*	4.72 ± 0.14**	1.73 ± 0.15**
L-SOD	1.52 ± 0.09**	2.20 ± 0.12*	4.50 ± 0.10**	1.87 ± 0.18**
SOD hydrolysate	2.66 ± 0.13	4.11 ± 0.07	2.47 ± 0.61	2.78 ± 0.24
Positive control	2.10 ± 0.09	2.31 ± 0.26	4.54 ± 0.14**	2.29 ± 0.28

The data are presented as mean ± SD, *n* = 6 each group. *P < 0.05, **P < 0.01 vs. model group.

Glycated albumin (GA) is the product of non-enzymatic glycation, a reaction between glucose and serum albumin in blood. GA is proportional to the concentration of blood glucose. Fasting plasma was collected from the rats after 4 weeks of intervention, and the content of plasma GA was also measured. It was found (Table 3) that the GA levels in the SOD group, the L-SOD group, and the positive control group were very significantly reduced, by 48.25%, 49.75%, and 33.30% (*P* < 0.01), respectively, compared with the level in the model group (795.84 μmol/L), which was very significantly higher than that in the normal group (427.61 μmol/L). However, there was no significant difference between the SOD hydrolysate group and the model group (*P* > 0.05).

**Table 3:** Fasting plasma glucose-related indexes of GA, GC, and AMPK.

Group	Glycated albumin (μmol/L)	Glucagon (pg/mL)	AMPK (ng/L)
Normal	427.61 ± 17.68**	236.06 ± 7.58**	583.12 ± 9.76**
Model	795.84 ± 5.53	434.85 ± 33.98	206.03 ± 8.06
SOD	411.80 ± 36.78**	153.69 ± 18.71**	329.45 ± 4.89**
L-SOD	399.87 ± 18.42**	135.52 ± 19.97**	336.85 ± 15.77**
SOD hydrolysate	776.51 ± 13.32	448.62 ± 31.29	226.05 ± 7.76
Positive control	530.81 ± 20.13**	195.09 ± 29.5**	240.71 ± 4.70*

The data are presented as mean ± SD, *n* = 6 each group. *P < 0.05, **P < 0.01 vs. model group.

**Fig. 4:**
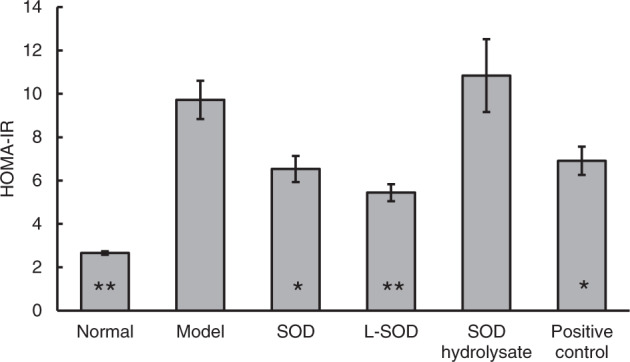
IR index in rats after 4-week intervention. The data are presented as mean ± SD, *n* = 6 each group. *P < 0.05, **P < 0.01 vs. model group.

T2D is especially characterized by glucose intolerance due to insufficient production of insulin in relation to IR^24^. We also confirmed (Fig. 4) that the IR index (HOMA-IR) of the model group was very significantly higher than that of the normal group, by 265.41% (*P* < 0.01). However, the HOMA-IR of the SOD group, L-SOD group, and positive control group decreased significantly or very significantly, by 32.82% (*P* < 0.05), 44.03% (*P* < 0.01), and 28.91% (*P* < 0.05), respectively, compared with the model group, after 4 weeks of administration.

The importance of glucagon (GC) for diabetic hyperglycemia of T2D has been previously demonstrated^24^. Adenosine monophosphate-activated protein kinase (AMPK) is an enzyme that plays an important role in insulin signaling and the metabolism of glucose and fats^25^. AMPK activation has been shown to be required for the glucose-lowering effect of metformin^26^. As shown in Table 3, compared with the normal group, GC in the model group increased by 84.21% (*P* < 0.01). However, the GC levels were significantly lower, by 64.66% (*P* < 0.01), 68.84% (*P* < 0.01), and 55.14% (*P* < 0.01) in the SOD group, L-SOD group, and positive group, respectively, than in the model group. The AMPK activity levels of the SOD group, L-SOD group, and positive control group increased 59.90% (*P* < 0.01), 63.50% (*P* < 0.01), and 16.83% (*P* < 0.05), respectively, compared with the model group, while the AMPK level of the model group was 64.67% (*P* < 0.01) significantly lower than in the normal group.

### Orally administered SOD samples reduced intestinal damage in T2D model rats

#### Recovery of colon density by SOD samples in T2D model rats

**Fig. 5:**
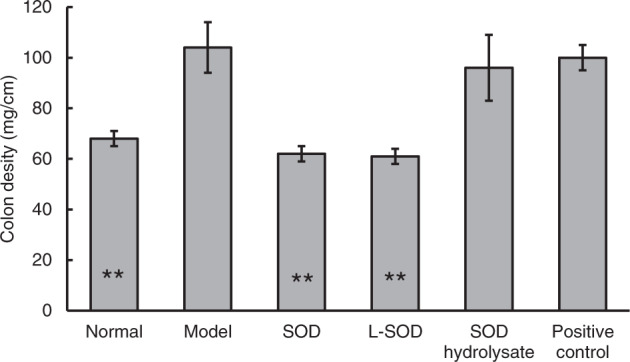
Colon density in rats after 4-week intervention. The data are presented as mean ± SD, *n* = 6 each group. *P < 0.05, **P < 0.01 vs. model group.

After 4 weeks of administration of SOD samples, colon density was determined, because increased intestinal density is a characteristic of intestinal inflammation^27^. As shown in Fig. 5, the colon density of the model group was very significantly higher than that of the normal group, by 52.94% (*P* < 0.01); the values in the SOD and L-SOD groups were significantly lower than in the model group, by 40.38% and 41.35%, respectively (*P* < 0.01). There was no significant difference in the SOD hydrolysate group or the metformin positive control group, when they were compared with the model group (*P* > 0.05).

#### Recovery of integrity of the intestinal barrier with SOD samples in T2D model rats

**Fig. 6:**
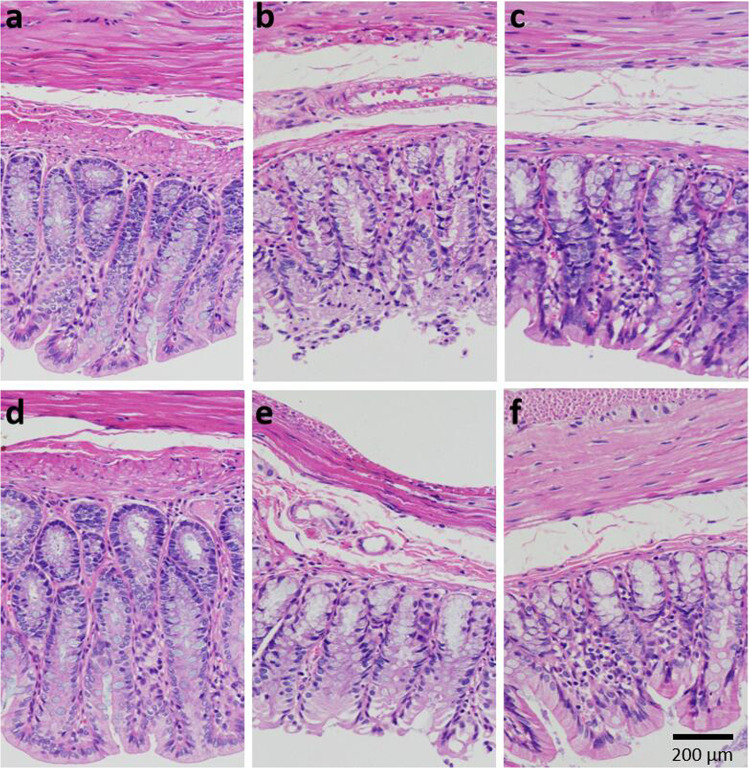
Colonic structure of rats after 4-week intervention. **a** Normal; **b** Model; **c** SOD; **d** L-SOD; **e** SOD hydrolysate; **f** Positive control.

The colonic tissues were taken for H&E staining to observe the integrity of the intestinal barrier and the sequential infiltration of inflammatory cells. As shown in Fig. 6, the colonic epithelium was intact and continuous, the cell edge clearly visible, the crypt structure intact and regular, the mucosa and lamina propria normal, and the muscle layer not unusual in the normal group (Fig. 6a). In the model group (Fig. 6b), the epithelium was extensively exfoliated, the glandular structure severely destroyed, and the crypt structure was deformed or had disappeared. Moreover, a large number of inflammatory cells were clearly infiltrated into the colonic submucosa. The appearance of the SOD hydrolysate group (Fig. 6e) was basically the same as that of the model group, but the measures of the colonic barrier in the SOD group (Fig. 6c) and the positive control group (Fig. 6f) improved in comparison to the model group. The improvements included intact and continuous epithelial cells, regularly arranged cells and glands, as well as relatively intact crypts and decreased infiltration of inflammatory cells in mucosa and submucosa. The colonic integrity and the infiltration of inflammatory cells in the L-SOD group (Fig. 6d) were close to those in the normal group, indicating their intestinal integrity levels were significantly improved compared with the model group.

#### Recovery of tight junctions with SOD samples in the intestines of T2D model rats

Immunohistochemical staining of the tight junction proteins occludin and ZO-1 in the rat colon was carried out to investigate further the improvement of the intestinal barrier by treatment with SOD samples. The intestinal barrier consists of intestinal epithelial cells, cell junctions, and the outer mucosal layer. Cell junctions consist of a variety of junction proteins, and occludin and ZO-1 form the basic structure of the tight junction. Their abnormal distribution can lead to abnormalities of tight junction structure and function, increased permeability of the intestinal barrier, and the occurrence of intestinal damage.

**Fig. 7:**
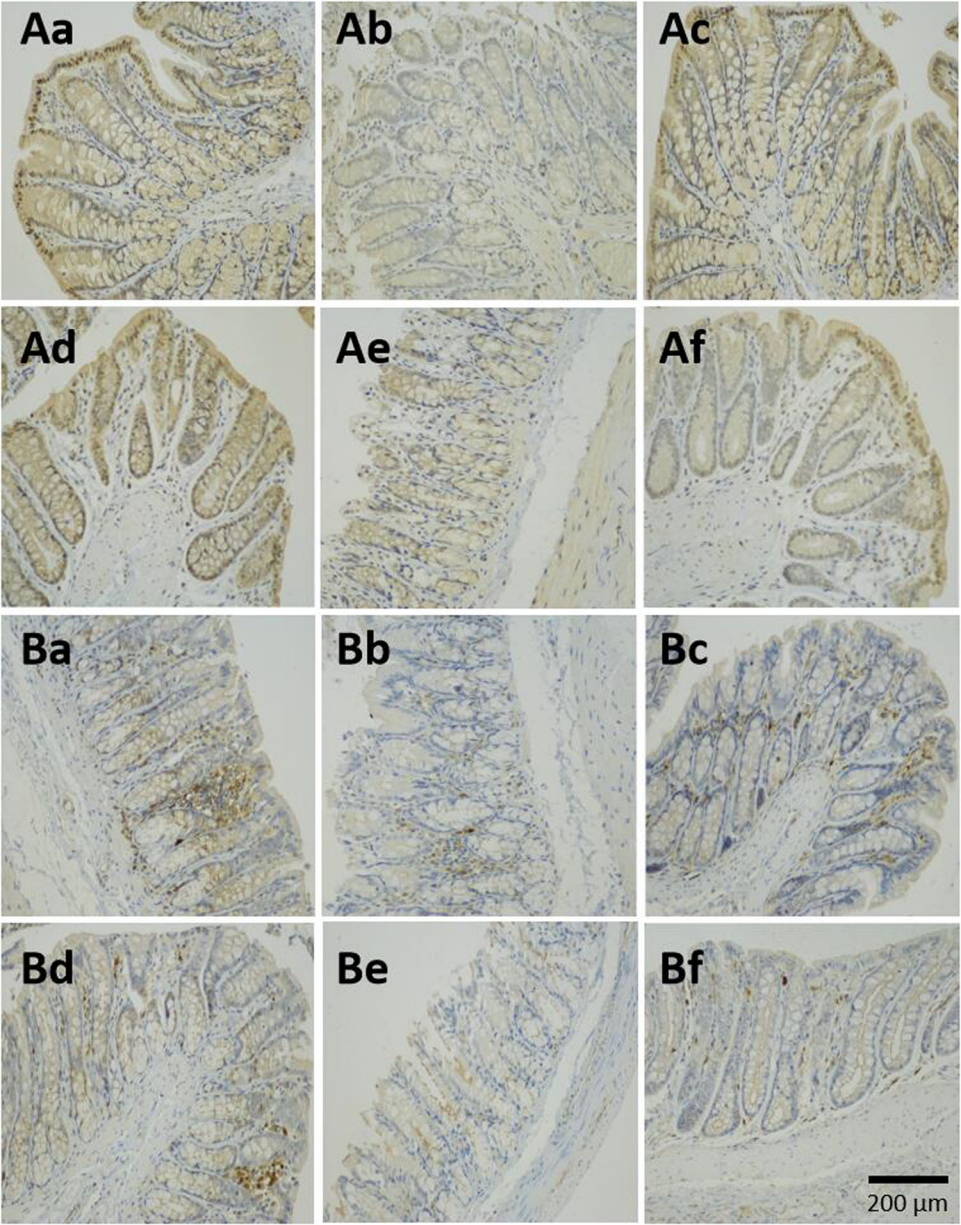
Immunohistochemical analysis of the tight junction proteins occludin and ZO-1 in the rat colon. **A** Occludin; **B** ZO-1; **a** Normal; **b** Model; **c** SOD; **d** L-SOD; **e** SOD hydrolysate; **f** Positive control.

Occludin locates at the apical side of intestinal epithelial cells and interweaves into a network to prevent increased intestinal permeability and protect the intestinal barrier. Occludin in the normal group (Fig. 7Aa) was evenly distributed and continuous, but in the model group (Fig. 7Ab), the occludin content decreased dramatically, with almost no tan occludin signal. The occludin content in the SOD group (Fig. 7Ac) recovered, close to that of the normal group; the L-SOD group Fig. 7Ad) was similar to the normal group, showing significant and continuous tan occludin signal, especially in the crypt; the content and continuity of occludin in the L-SOD group were even better than those in SOD group. The SOD hydrolysate group (Fig. 7Ae) was similar to the model group, with the content of occludin sharply reduced, and the zigzag distribution was interrupted. The content of occludin in the positive control group (Fig. 7Af) was slightly recovered and more continuous, but the content was less than that in the SOD group and the L-SOD groups.

ZO-1 localizes within intestinal epithelial cells. As shown in the B series of Fig. 7, ZO-1 in the normal rats is abundant and widely distributed, and the change trends of ZO-1 in each group of oral administration were similar to those of occludin in the A series of Fig. 7.

It was reported that superoxide-induced oxidative stress induced cellular redistribution of the occludin-ZO-1 complex in Caco-2 cells, a cell line of intestinal epithelium^28^. Therefore, these results are consistent with the previous report that the tight junction proteins occludin and ZO-1 were disturbed by the oxidative stress, and indicated that orally administered SOD showed effective recovery when there had been an abnormal distribution of occludin and ZO-1 with dysfunction of the tight junctions in the intestinal barrier.

### SOD samples decreased the systemic influx of LPS and inflammatory cytokines IL-1β, TNF-α, and IL-4 in fasting plasma of T2D model rats

**Fig. 8:**
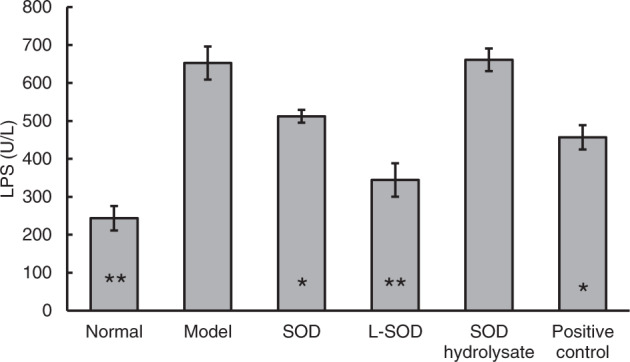
LPS level in the fasting plasma of normal or T2D model rats after 4-week intervention. The data are presented as mean ± SD, *n* = 6 each group. *P < 0.05, **P < 0.01 vs. model group.

LPS in human serum associates with IR^10^; therefore, the values of fasting plasma LPS in T2D model rats after 4-week of intervention were measured. As shown in Fig. 8, compared with the normal group, LPS in the model group increased by 168.08% (*P* < 0.01). However, the LPS levels in the SOD group, L-SOD group, and positive control group were significantly lower than in the model group, by 21.49% (*P* < 0.05), 47.21% (*P* < 0.01), and 29.99% (*P* < 0.05), respectively. There was no significant difference between the L-SOD group and the normal group (*P* > 0.05), but the difference between the L-SOD group and the SOD group was significant (*P* < 0.05).

LPS-induced inflammation causes elevation of the inflammatory cytokines IL-1β, TNF-α, and IL-4^29^. The data in Table 4 indicated that IL-1β, TNF-α, and IL-4 in the model group significantly increased, by 252.25% (*P* < 0.01), 84.67% (*P* < 0.05), and 112.12% (*P* < 0.01), respectively, compared with the normal group. However, compared with the model group, there were significant decreases, of 28.70% (*P* < 0.05), 42.77% (*P* < 0.01), and 54.09% (*P* < 0.01) in the IL-1β of the SOD group, L-SOD group, and positive control group, respectively. The TNF-α of the L-SOD group decreased by 37.55% (*P* < 0.05), and there were significant decreases, of 38.21% (*P* < 0.05) and 45.71% (*P* < 0.05) in the IL-4 in the SOD group and L-SOD group, respectively.

**Table 4:** Inflammatory cytokines IL-1β, TNF-α, and IL-4 in fasting plasma of T2D model rats.

Group	IL-1β (pg/mL)	TNF-α (pg/mL)	IL-4 (pg/mL)
Normal	196.94 ± 11.20**	1.37 ± 0.14*	2.64 ± 0.29**
Model	693.73 ± 43.46	2.53 ± 0.41	5.60 ± 0.72
SOD	494.62 ± 76.88*	1.63 ± 0.19	3.46 ± 0.49*
L-SOD	396.99 ± 49.75**	1.58 ± 0.10*	3.04 ± 0.39*
SOD hydrolysate	624.82 ± 23.59	2.63 ± 0.30	4.51 ± 0.35
Positive control	318.47 ± 61.19**	1.61 ± 0.24	4.18 ± 0.38

The data are presented as mean ± SD, *n* = 6 each group. *P < 0.05, **P < 0.01 vs. model group

These data indicated that LPS concentration in the fasting serum of the model group was significantly higher than in the normal group, and it caused the increase of the inflammatory cytokines IL-1β, TNF-α, and IL-4. The improvement of these indexes in the three groups of SOD samples was different: L-SOD was better than SOD, and SOD hydrolysate had little effect on improvement.

**Fig. 9:**
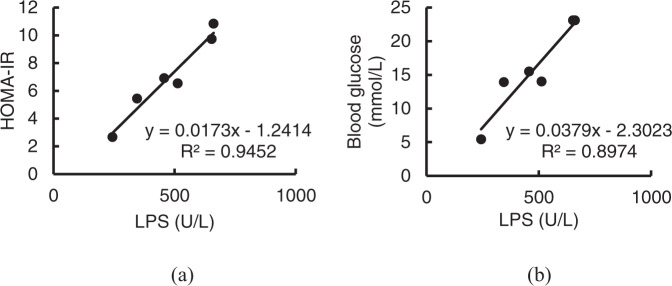
Relevance between HOMA-IR or blood glucose and LPS. **a** HOMA-IR; **b** Blood glucose.

The relationships between IR index or blood glucose and LPS were calculated according to the data in Figs. 3, 4, and 8. It was found that the IR index and the blood glucose exhibited positive relationships with LPS, supported by R^2^ values of 0.9452 and 0.8974, respectively (Fig. 9). This result is consistent with the well-known hypothesis that lowering plasma LPS concentration could be a potent strategy for the control of diabetes^30^.

## Discussion

**Fig. 10:**
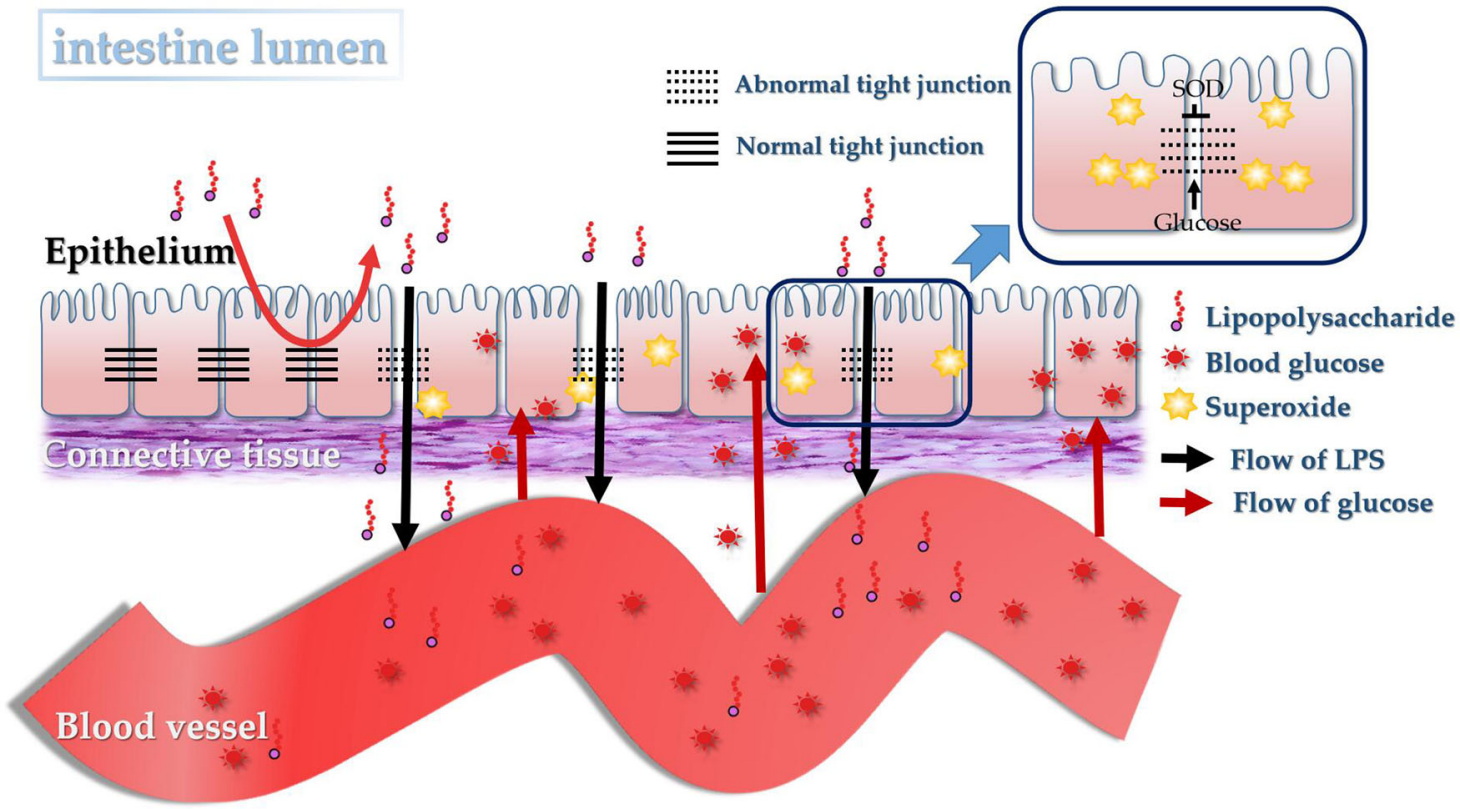
SOD inhibits hyperglycemia-induced oxidative damage to intestinal barrier integrity. Persistent vascular transport of glucose into intestinal epithelial cells mediates an elevated level of superoxide. This results in increased intestinal permeability via oxidative damage to tight junction-related intestinal barrier integrity, permissive translocation of LPS into systemic circulation, chronic inflammation, and IR in T2D. However, oral administration of intestine-targeted SOD can reverse the consequent loss in barrier integrity and systemic influx of LPS, thereby decreasing IR and blood glucose.

The data in Figs. 1, 2 and Table 1 indicated obvious oxidative stress in the intestinal tract, especially in the colons of T2D rats; orally administered SOD effectively reduced the oxidative stress, and the order of the effects of these SOD preparations was L-SOD > SOD > SOD hydrolysate. MDA is a physiologic ketoaldehyde produced by peroxidative decomposition of unsaturated lipids, and GSSG/GSH is the ratio of GSSG (oxidized glutathione) to GSH (reduced glutathione). Although both indexes reflect the state of oxidative stress, GSSG/GSH is likely more sensitive than MDA to the status shift of oxidative stress, as shown in the monocytes of a previous report^31^. This would be the reason why L-SOD could reducing GSSG/GSH better than SOD, but MDA as almost the same as SOD. As shown in Fig. 1, the MDA content in colons of the SOD group of T2D rats (0.69) was not significantly higher than in the L-SOD group (0.62) (*P* > 0.05), while the GSSG/GSH ratio (Fig. 2) of the SOD group (1.63) was significantly higher than that of the L-SOD group (0.95) (*P* < 0.01). We think it is likely that this oxidative stress of the intestine disrupts the occludin-ZO-1 complex-associated intestinal barrier and thus increases systemic influx of LPS (Fig. 8), leading to chronic inflammation-associated IR (Fig. 9a), a typical feature of T2D, and subsequent hyperglycemia (Fig. 9b) and related indexes (Figs. 3, 4, Tables 2, and 3). However, we don’t have data of directly measuring glycosylated hemoglobin levels in each group but did determine glycated albumin to better express the level of blood glucose of T2D model rats during the 4-week intervention of our study. Because glycated albumin data can reflect the average glucose level within 3-week, but HbA1c is measured primarily to determine the 12-week average blood glucose level^32,33^. Orally administered SOD samples, especially L-SOD, were able to decrease oxidative stress-induced intestinal damage significantly (Figs. 5–7) and to decrease the systemic influx of LPS (Fig. 8), thereby reducing inflammatory cytokines (Table 4), increasing AMPK activity (Table 3) and insulin sensitivity (Fig. 9a), and then significantly reducing blood glucose (Fig. 9b). Therefore, these results confirmed, at least in part, the glucose-lowering effect of orally administered SOD in diabetes via a decrease in oxidative damage as well as a decrease in the systemic influx of LPS in the intestine (Fig. 10). This mode of action of SOD is similar to that of curcumin, which maintains the integrity of the intestinal barrier function as a mechanism of attenuating metabolic diseases (diabetes, atherosclerosis, kidney disease)^11^. These results support the notion that food bioactives can restore intestinal barrier function, potentially to reduce the risk for disease^6^.

It is notable that in this work natural SOD exhibited a substantial glucose-lowering effect, although the effect was inferior to that of L-SOD in T2D model rats (Fig. 3). This capacity of natural SOD might result from small amounts of natural SOD surviving as enzymatic active molecule from the stomach into the intestine^20^. This possibility may also explain our previous finding that orally administered SOD substantially decreased blood glucose concentration in alloxan-induced diabetic rats^21^. We are trying to test this possibility with SOD labeled with fluorescein isothiocyanate (FITC).

L-SOD used in this work was composed of bovine blood Cu/Zn SOD, lecithin, and cholesterol. It was previously suggested that the oral bioavailability of SOD and L-SOD was very low in blood, because of its high molecular weight and the low pH and high proteolytic activity in the digestive tract; SOD and L-SOD, although containing a foreign protein, were well tolerated and produced no acute or delayed toxic effects in human^18,34^. This composition makes it usable in the functional food industry, although the dosage of the composited lecithin in the L-SOD is too low to exert substantial glucose-lowering activity, compared to the much higher dosage of lecithin used in previous reports on diabetic model rats^35,36^.

Compared with the typical antidiabetic drug metformin, L-SOD in this work showed superior effects on the control of blood glucose (Fig. 3) and lipids (Table 2). It is notable that metformin is more commonly associated with gastrointestinal adverse effects than most other antidiabetic medications^37^. Moreover, the improvements due to L-SOD on LPS and its related indexes were better than those due to metformin in the positive control group (Fig. 8 and Table 4); this may be related to the different mechanisms of actions of SOD and metformin. It was reported previously that metformin effectively reduced endogenous glucose production and decreased mitochondrial redox states after adsorption into blood, but did not directly target the intestinal barrier^38^. Therefore, this work suggests that orally administered SOD could be a novel glucose-decreasing approach different than metformin, and further shows that the intestinal barrier, with its oxidative stress, may be a target for potential prevention and amelioration of diabetes mellitus.

However, more work should be carried out, such as a study of the effect of SOD on microbiota. It has been suggested that microbiota dysbiosis contributes to the onset and maintenance of IR and glycemic control^39^, and changes in the diversity and abundance of microbiota possibly affect the production of LPS inside the intestinal lumen, thereby regulating the systematic influx of LPS^10^. In addition, it was recently reported that oxidative stress changed the diversity and abundance of microbiota^40,41^. Furthermore, the inhibitory effect of SOD on the cellular redistribution of occludin-ZO-1 complexes may rely on a tyrosine-kinase dependent mechanism, which can be confirmed on the Caco-2 cell line^28^. It would be attractive to investigate the downstream effect of SOD-induced recovery of the intestinal barrier on the secretion of enterohormones, such as GC-like peptide-1 and ghrelin, which control glucose homeostasis via the brain-gut axis^42^. We should also consider studying the effect of orally administered SOD on genetic or sole diet-induced diabetes models, so as to clarify whether or not SOD simply ameliorates the oxidative stress of STZ intoxication. Moreover, SOD intervention should be further investigated to optimize the improvement of diabetes mellitus. Optimization is needed, because on the one hand, we think that the minimal effective enzymatic activity is quite low (based on the substantial effect in the SOD group); but on the other hand, even after a 4-week administration, the blood glucose in the L-SOD group was still significantly higher than in the normal group, and it reached a plateau after 16 days (Fig. 3). Figure 3 shows none of Model, SOD, L-SOD, positive control and SOD hydrolysate groups could achieve the same blood glucose levels comparable to Normal group. That is likely because of their high fat and high carbohydrate diet used in the groups of Model, SOD, L-SOD, positive control and SOD hydrolysate, different from that in Normal group. This would imply that the glucose-lowering effects of orally administered SOD, similar to the effects of metformin and other anti-hyperglycemic drugs, may be further improved with the control of caloric intake^43,44^ or diet modification^45^.

## Methods

### Experimental design

**Fig. 11:**
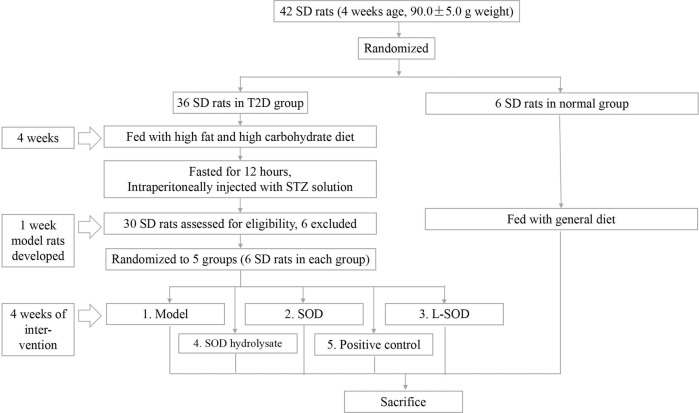
Study flow chart. Development, grouping, and intervention of T2D model rats.

As described below, a T2D rat model was first produced to mimic the oxidative stress in the intestine and the antioxidant effect of SOD. These effects were observed by measuring MDA (malondialdehyde); the ratio of oxidized glutathione (GSSG) to reduced glutathione (GSH), i.e., GSSG/GSH; and the antioxidant enzymatic activity of SOD, catalase (CAT), and glutathione peroxidase (GPx). Subsequently, T2D rats were randomly divided into five groups, as shown in Fig. 11, and orally administered with natural SOD (SOD group), L-SOD (L-SOD group), SOD hydrolysate (SOD hydrolysate group), normal saline (model group), or metformin (positive control group), to compare with the data of the normal group, which did not have diabetes but received normal saline by oral gavage. Fasting serum indexes, including blood glucose, glycated albumin (GA), insulin, blood lipids, glucagon (GC), adenosine monophosphate-activated protein kinase (AMPK), LPS, and inflammatory cytokines, were measured, and measurement of colon density, H&E staining, and immunohistochemical analysis of tight junction proteins in the colon were carried out. Finally, blood glucose concentration and related indexes were analyzed along with intestinal damage and related data, as well as LPS and inflammatory cytokines.

### Materials

Bovine blood Cu/Zn SOD was provided by the Institute of Life Sciences, Tianjin, China, with a specific enzymatic activity of 40,000 U/mg, measured according to the indirect assay of hydroxylamine hydrochloride^46^. Metformin hydrochloride was from Tianan Pharmaceutical Co., Ltd., Guizhou, China. Streptozotocin (STZ, purity ≥98%) purchased from Sigma-Aldrich was dissolved in citric acid buffer (pH 4.2–4.5) with a concentration of 10.0 mg/mL, and the solution was freshly prepared just before use. A blood glucose meter and matching test paper (On call EZIII) were supplied by ACON Laboratories, Hangzhou, China. ELISA kits for insulin, GC, LPS, and GA, as well as the AMPK kit were purchased from Enzyme Standard Biotechnology Co., Ltd., Jiangsu, China. Kits for MDA, blood lipid, GSSG, reduced GSH, CAT, and GPx were from Nanjing Jiancheng Bioengineering Institute, China. Eosin, hematoxylin solution, antigen-retrieval solution, antibodies to occludin, IL-1β, TNF-α, and IL-4, were provided by Servicebio Technology Co., Ltd., Wuhan, China. ZO-1 polyclonal antibody was obtained from Thermo Fisher Scientific.

A sample of natural SOD, bovine blood Cu/Zn SOD, was prepared by dissolving 40.5 mg of bovine blood Cu/Zn SOD in 180.0 mL of normal saline. A sample of liposome SOD, abbreviated as L-SOD, was prepared from bovine blood Cu/Zn SOD, lecithin, and cholesterol. Briefly, 50.0 mg of bovine blood Cu/Zn SOD in a centrifuge tube was added to 10.0 mL phosphate buffer (pH 7.40, 0.20 M) and shaken gently to dissolve it. Then the solution was poured into a centrifuge tube containing 200.0 mg lecithin and 50.0 mg cholesterol, together with 30.0 mL of diethyl ether. After shaking gently, the mixture was treated with an ultrasonic oscillator for 5 min. The treated solution was then poured into a 100.0-mL flask and evaporated with a rotary evaporator at 40 °C without light for 10 min to remove the organic phase fully. The prepared emulsion was centrifuged at 10,000 g for 5 min, to obtain the supernatant as the sample of L-SOD. This L-SOD was confirmed to still retain 85.84% or 94.75% of its enzymatic activity, after being treated for 4 h in either simulated gastric fluid or simulated intestinal fluid, respectively^47^. This result indicated the L-SOD was tolerant to digestive enzymes. Then it was kept at 4 °C, avoiding light by covering with foil, for subsequent applications.

SOD hydrolysate was prepared according to the following procedure. Forty-five milliliters of normal saline with 40.5 mg of bovine blood Cu/Zn SOD was uniformly mixed with 135.0 mL of artificial gastric juice (containing 2.0 g NaCl, 7.0 mL HCl, pH 1.2, and 3.2 g pepsin in 1000 mL), and then incubated at 37 °C overnight. The hydrolysate solution was identified by SDS-PAGE and enzyme activity analysis to have no intact SOD or detectable enzyme activity. This solution was kept at 4 °C for subsequent applications.

### Development, grouping, and intervention of T2D model rats

As shown in Fig. 11, 42 male SD rats weighing 90.0 ± 5.0 g were purchased from Zhejiang Laboratory Animal Center, Zhejiang Academy of Medical Sciences (Hangzhou, Zhejiang, China, SCXK(Zhe)2019-0002). Six were randomly selected for a normal group and were fed a general diet. The remaining 36 were fed with a high-fat and high-carbohydrate diet to develop T2D model rats^48^. One month later, these 36 rats were fasted for 12 h and were then intraperitoneally injected with STZ solution with a dosage of 40.0 mg/kg body weight. They were then fed 1 h after injection. Blood glucose was detected on the third and seventh days after the injection, and rats with stable fasting blood glucose higher than 16.7 mmol/L were selected as T2D model rats. From the group, 30 rats were successfully made diabetic.

During the next 4 weeks of intervention, the model rats were continually fed with the high-fat and high-carbohydrate diet and randomly divided into five groups according to body weight and blood glucose, with six rats in each group. Among them, the positive control group was fed by gavage with 10.0 mg/kg of metformin hydrochloride; the SOD group and L-SOD group were fed with 30,000 U/kg body weight of the sample of bovine blood Cu/Zn SOD or L-SOD, respectively; the SOD hydrolysate group was fed with the solution of SOD hydrolysate by gavage, with the same weight of bovine blood Cu/Zn SOD as the SOD group; and the model group was treated by gavage with the same amount of normal saline (3.0 mL/Kg) as the normal group. Each regular daily administration was at 15:00. Every 4 days, blood glucose and body weight were measured, and dosages were adjusted according to body weight.

All animals used in this study were treated according to the Guide for the Care and Use of Laboratory Animals published by the National Institutes of Health. The protocol was approved by the Animal Experiment Ethics Committee of Zhejiang Academy of Medical Sciences (approved protocol ID: 2020R043002). All surgical procedures were performed under inhalation anesthesia using isoflurane, and every effort was made to reduce suffering. All animals were sacrificed with deep anesthesia and bleeding caused by cutting the inferior vena cava.

### Sample preparation and measurement

#### Preparation of the tissue homogenate of colon

A colon sample was added to nine times its mass of normal saline and then transferred into a homogenizer to make a tissue homogenate. Subsequently, the homogenate was centrifuged at 3000 rpm, 4 °C, for 15 min. The supernatant was taken as the sample of 10.0% tissue homogenate. The 10% homogenate sample was diluted with normal saline at a ratio of 1:9 to prepare a sample of 1.0% tissue homogenate.

### Determination of the level of MDA, GSSG, and GSH, as well as enzymatic activity of SOD, CAT, and GPx

A sample of 10.0% tissue homogenate was used for the determination of MDA content and SOD activity in colon tissue, and 1.0% tissue homogenate was used for measuring CAT, GPx, GSH, and GSSG.

The concentrations of MDA, GSSG, GSH and SOD, CAT, GPx activity were analyzed with colorimetric assay kits (Nanjing Jiancheng Bioengineering Institute, Nanjing, China).

### Measurement of blood glucose, insulin, GC, GA, LPS, enzymatic activity of AMPK, and blood lipid level

Blood glucose was determined by blood glucose meter with tail tip blood of rats after fasting for 6 h.

Concentrations of insulin, GC, GA, and LPS, and enzymatic activity of AMPK, were measured after fasting plasma was prepared by centrifugation at 3000 rpm, 4 °C for 15 min with colorimetric assay kits (Enzyme Standard Biotechnology Co., Ltd., Jiangsu, China). Blood lipid level in fasting plasma was determined with colorimetric assay kits (Nanjing Jiancheng Bioengineering Institute, China).

### Calculations of IR index and colonic density

The homeostatic model assessment (HOMA) method is used to quantify IR. The IR index of HOMA-IR was determined according to the values of the concentration of insulin and blood glucose in fasting plasma measured by the above procedures, and was calculated as follows:

IR index = (concentration of insulin×concentration of blood glucose)⁄22.5

Approximately 10 cm of the colon was sampled from 2 cm below the ileocecal valve to 2–3 cm above the anus after the rat abdominal cavity was opened; the weight and length were measured immediately. During sampling, the integrity of colon tissue was not destroyed by pulling, ensuring the length of the tissue itself. Then, colon density was calculated as follows:

Colonic denisity = (colonic weight(mg))⁄(colonic length(cm))

### H&E staining of colon tissue

A portion of the colon was fixed by paraformaldehyde for more than 24 h; then the appropriate block of tissue was prepared and dehydrated to be transparent before being embedded in paraffin. Subsequently, a section of 7.0 μm thickness cut by a paraffin slicer was placed on the slide and moved to the oven for dewaxing. Then the slice was dyed with hematoxylin for 1 min and eosin for 5 min. Finally, the colon structure was observed under the microscope after being dehydrated to transparency and sealed with neutral gum.

### Immunohistochemical analyses of tight junction proteins occludin and ZO-1 in colon

After the slices for these analyses were prepared according to the same procedure as for H&E staining, the antigens were retrieved. The retrieval method involved putting slices into the retrieval box, adding antigen-retrieval solution, heating in a pressure cooker for 2 min, cooling naturally after automatically depressurizing and removing from the heat source, discarding the retrieval solution, and finally rinsing with PBS buffer. Next, the slices were treated with 0.5% Triton X-100 for 20 min at room temperature. Then the slices were transferred into a wet box with 3.0% hydrogen peroxide to eliminate the enzymatic activity of endogenous peroxidase. Subsequently, glass slides were treated with a solution of the antibodies against occludin or ZO-1 after blocking the slices with normal goat serum at room temperature for 30 min, and then they were placed in the wet box and incubated overnight at 4 °C. Next, the solution of secondary antibody was added, and slides were incubated at room temperature for 1 h before full rinsing with PBS buffer. Then, the slices underwent sequential treatment by DAB staining for 10 min, PBS buffer rinsing for 1 min, hematoxylin re-staining for 3 min, differentiation with hydrochloric acid alcohol, returning to blue color by ammonia treatment, PBS rinsing again for 1 min, dehydrating to transparency, and sealing with neutral gum. Finally, they were observed under a microscope.

### Measurement of inflammatory cytokines IL-1β, TNF-α, and IL-4 in fasting plasma

These three inflammatory cytokines were measured by the immunochemical method with matching antibodies (Servicebio Technology Co., Ltd., Wuhan, China).

### Statistical analysis

All data are presented as the mean ± standard deviation of the mean (SD). For two groups, an unpaired two-tailed *t*-test was performed for intergroup comparisons. For more than two groups, one-way analysis of variance (ANOVA) and the post hoc multiple comparisons (LSD method) were used for intergroup comparisons. A *P* value of less than 0.05 was considered statistically significant.

## Data Availability

The authors declare that the data supporting the findings of this study are available within the article.
